# *Gtsf1* is essential for proper female sex determination and transposon silencing in the silkworm, *Bombyx mori*

**DOI:** 10.1371/journal.pgen.1009194

**Published:** 2020-11-02

**Authors:** Kai Chen, Ye Yu, Dehong Yang, Xu Yang, Linmeng Tang, Yujia Liu, Xingyu Luo, James R. Walter, Zulian Liu, Jun Xu, Yongping Huang

**Affiliations:** 1 Key Laboratory of Insect Developmental and Evolutionary Biology, Center for Excellence in Molecular Plant Sciences, Shanghai Institute of Plant Physiology and Ecology, Chinese Academy of Sciences, Shanghai, China; 2 CAS Center for Excellence in Biotic Interactions, University of Chinese Academy of Sciences, Beijing, China; 3 Department of Ecology and Evolutionary Biology, University of Kansas, NV, United States of America; New York University, UNITED STATES

## Abstract

Sex determination pathways are astoundingly diverse in insects. For instance, the silk moth *Bombyx mori* uniquely use various components of the piRNA pathway to produce the *Fem* signal for specification of the female fate. In this study, we identified BmGTSF1 as a novel piRNA factor which participates in *B*. *mori* sex determination. We found that *BmGtsf1* has a distinct expression pattern compared to *Drosophila* and mouse. CRISPR/Cas9 induced mutation in *BmGtsf1* resulted in partial sex reversal in genotypically female animals by shifting expression of the downstream targets *BmMasc* and *Bmdsx* to the male pattern. As levels of *Fem* piRNAs were substantially reduced in female mutants, we concluded that *BmGtsf1* plays a critical role in the biogenesis of the feminizing signal. We also demonstrated that BmGTSF1 physically interacted with BmSIWI, a protein previously reported to be involved in female sex determination, indicating BmGTSF1 function as the cofactor of BmSIWI. *BmGtsf1* mutation resulted in piRNA pathway dysregulation, including piRNA biogenesis defects and transposon derepression, suggesting *BmGtsf1* is also a piRNA factor in the silkworm. Furthermore, we found that *BmGtsf1* mutation leads to gametogenesis defects in both male and female. Our data suggested that *BmGtsf1* is a new component involved in the sex determination pathway in *B*. *mori*.

## Author summary

Sex determination is a fundamentally important process in most sexually reproducing metazoan. Nevertheless, the underlying mechanisms of sex determination are highly diverse. In *B*. *mori*, piRNAs derived from the W-chromosome-linked *Fem* precursor serve as the primary female determining signal. However, we still know little about the initiation of *B*. *mori* sex determination and its relationship with piRNA pathway. Here, we provided evidence that BmGTSF1 is a novel piRNA factor which is indispensable for *B*.*mori* female sex determination. Mutations in *BmGtsf1* resulted in dysregulation of the piRNA pathway and caused partial female-male sex reversal. We also detected dramatic diminution of *Fem* piRNA in female mutant, indicating BmGTSF1 regulates *B*. *mori* sex determination via piRNA pathway. More importantly, we showed that BmGTSF1 interacted with BmSIWI, which protein had been reported to be involved in piRNA pathway and sex determination in *B*. *mori*, supporting the conclusion that BmGTSF1 is a novel factor for piRNA pathway and sex determination.

## Introduction

The mechanisms of sex determination are highly diverse in different species of insects [1–4]. In *Drosophila melanogaster*, the initial signal of sex is the X chromosome dosage present in the early zygote [5–8]. Individuals with two X chromosomes (XX) will adopt the female fate, while individuals with one X chromosome (XY) will develop to be male [8–12]. Different from XX/XY systems, the silkworm, *B*. *mori* possesses the WZ/ZZ sex determination system in which females are the heterogametic sex [13, 14]. A PIWI-interacting RNA (piRNA) precursor called *Feminizer* (*Fem*), which has more than 30 copies distributed in the sex determination region of the W chromosome, has been proposed to act as the female determining factor in the silkworm. piRNAs derived from *Fem* repress the expression of the *Masculinizer* gene (*Masc*) which located on the Z chromosome through piRNA pathway, a mechanism for transposon silencing. When *Masc* levels are low, the final effector in the pathway, the *dsx* gene, expresses female-specific variants which instruct female development [15, 16]. In contrast, when *Fem* is absent in ZZ individuals, *Masc* is expressed at high levels and activates expression of male-specific variants of *dsx* resulting in male development. Except for *Fem* and *Masc*, there are other two sex determination factors, *B*. *mori insulin-like growth factor II mRNA-binding protein* (*BmIMP*) and *P-element Somatic Inhibitor* (*BmPSI*). *BmPSI* and male-specific *BmIMP* (*BmIMP*^*M*^) are involved in male-specific *Bmdsx* splicing through inhibiting the female-specific *Bmdsx* splicing in males [17, 47].The function of *Fem* requires assistance of piRNA pathway gene like *BmSiwi*, deletion of which can cause partial sexual reversal of female [18]. However, except for BmSIWI and its cofactor BmASH2, no any other piRNA pathway factor was shown to be involved in sex determination pathway in the silkworm.

In the past few years, many novel piRNA factors have been uncovered. Several studies have shown that an evolutionarily conserved protein called Gametocyte-specific factor1 (GTSF1) is involved in the piRNA pathway [19–22]. GTSF1 is a protein which consists of two U11-48K-like CHHC-type Zinc finger motifs at N-terminal and a C-terminal end without discernable domains [20, 21]. In *Drosophila* and mouse, GTSF1 is preferentially expressed in germ cells. In *Drosophila*, GTSF1 (also known as Asterix) is required for female fertility and interacts with PIWI via its C-terminal end. DmGTSF1 is essential for transposon silencing but not for piRNA biogenesis. In absence of GTSF1, localization of PIWI is unaffected, and PIWI can still bind piRNAs, whereas introduction of H3K9me3 marks into transposon loci is impacted [20, 21]. In mouse, GTSF1 is necessary for spermatogenesis and male fertility, and associates with PIWI proteins via its central region instead of its C-terminal tail. Though murine GTSF1 is indispensable for secondary piRNA genesis, it is not needed for primary piRNA biogenesis [22].

Silencing transposable elements (TEs) is the primary function of PIWI-interacting RNA (piRNA) pathway, despite it also plays vital role in the silkworm sex determination. TEs pose a potential threat to genome stability. In particular, their presence in germ cells can cause damage to the germline genome and may result in sterility [23–25]. In the germline, piRNA pathway functions as a defense system in silencing TEs, and maintains genomic integrity [26–28]. piRNAs are a class of small non-coding RNA with 24–32 nucleotides deeply conserved from yeast to human. piRNAs bind to PIWI proteins and form piRNA induced silencing complex (pi-RISC) to repress TEs expression at the transcriptional and post-transcriptional level [29–31]. The biosynthesis of piRNAs consists of two pathways: primary processing pathway and secondary processing pathway (also referred to as the ping-pong cycle) [25, 27]. In *Drosophila*, three PIWI proteins serve as the key components of piRNA pathway: Piwi, Argonaute (Ago3) and Aubergine (Aub). PIWI and Aub bind to primary piRNAs, while Ago3 binds to secondary piRNAs [32–34]. In *B*. *mori*, there are only two PIWI proteins: BmSIWI and BmAgo3 [35, 36], indicating discrepant mechanisms of piRNA pathway among species. Beyond PIWI proteins, many other factors are also indispensable for piRNA pathway, and mutational disruption of these piRNA factors can result in spermatogenesis or oogenesis defects in mouse and *Drosophila* respectively [26, 31, 37–43]. However, the research upon piRNA pathway in the silkworm were concentrated on BmN4 cell line, thus, little was known about the function of piRNA pathway in the silkworm gametogenesis.

In this study we examined the hitherto unknown functions of the silkworm ortholog of *Gtsf1* (*BmGtsf1*). Of particular interest was our finding that BmGTSF1 plays a critical role in female sex determination. *BmGtsf1* mutations resulted in partial sex reversal of female animals, while sexual development of males remain unaffected. Furthermore, BmGTSF1 appears necessary for TE silencing during gametogenesis in both male and female, given that the lack of BmGTSF1 causes sterility most likely due to genomic instability. Subsequently, we found that BmGTSF1 interacted with BmSIWI (Silkworm PIWI protein), and that disruption of BmGTSF1 function led to transposon derepression and dysregulation of piRNA biogenesis. Taken together, our work reaveals that BmGTSF1 is a novel factor in female sex determination and piRNA pathway.

## Results

### Expression pattern of *BmGtsf1* and mutants construction

**Fig 1:**
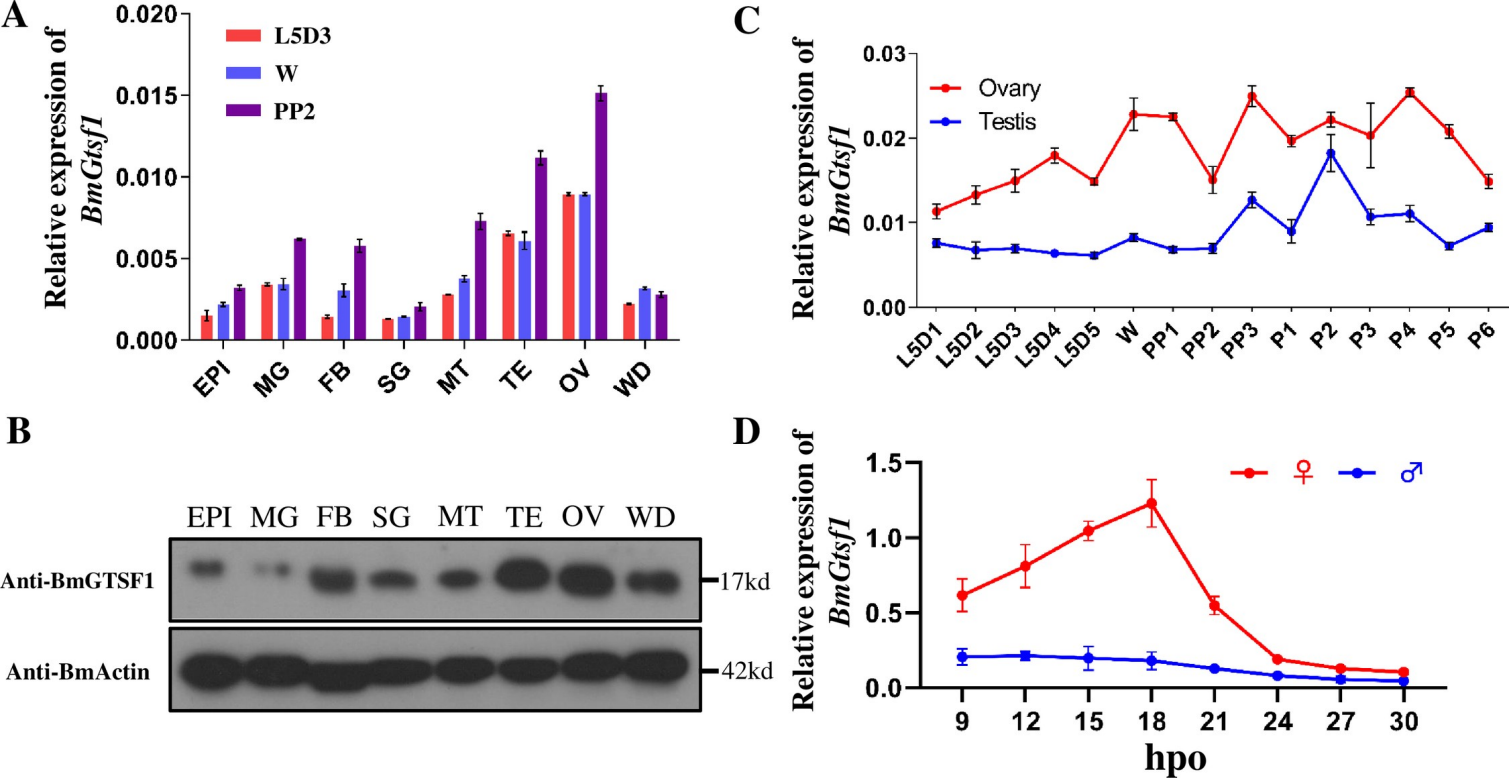
Expression patterns of BmGTSF1. (A) qRT-PCR analysis showed relative mRNA levels of *BmGtsf1* in eight tissues at three different stages: third day of fifth larval stage (L5D3), wandering stage (W), and day 2 of the prepupal stage (PP2). The mRNA levels were normalized to levels of *B*. *mori ribosomal protein 49* (*Bmrp49*). Error bars represent ± SD. (B) Western blot analysis of BmGTSF1 in different tissues in wandering stage. (C) The mRNA levels of *BmGtsf1* in testis and ovary from day 1 of fifth larval stage (L5D1) to day 6 of pupal stage (P6). Tissues tested were epidermis (EPI), midgut (MG), fat body (FB), silk gland (SG), malpighian tubule (MT), testis (TE), ovary (OV), and wing disc (WD). (D) Relative mRNA level of *BmGtsf1* from 9hpo to 30hpo (hours after oviposition).

Previous studies in *Drosophila* and mouse reported that *Gtsf1* is predominantly expressed in the germ line [20–22]. In the silkworm we found that *BmGtsf1* was ubiquitously expressed at all stages tested with a peak level of expression in gonads (Fig 1A and 1B). High levels of *BmGtsf1* mRNA present in gonads from early larval stage to pupal stage suggested an important role of *BmGtsf1* in gonadal development. In addition, the expression level of *BmGtsf1* in ovaries was higher than that in testes, revealing *BmGtsf1* may have more important function in female (Fig 1C). Moreover, we also found that the mRNA level of *BmGtsf1* in female was higher than that in male at early embryonic stage (Fig 1D).

To explore the physiological function of *BmGtsf1*, we utilized a previously described binary transgenic CRISPR/Cas9 system [44, 45] to establish somatic mutant lines (*ΔBmGtsf1*). Briefly, we constructed two transgenic silkworm lines expressing Cas9 and the sgRNA, respectively. The two lines were crossed to obtain somatic *BmGtsf1* mutants. We were able to generate lesions in the *BmGtsf1* locus in *ΔBmGtsf1* animals (S2A Fig and S2B Fig), and the depletion efficiency of RNA and protein levels was further confirmed by qRT-PCR and western blot (S2C Fig and S2D Fig).

### *BmGtsf1* is involved in female sex determination

**Fig 2:**
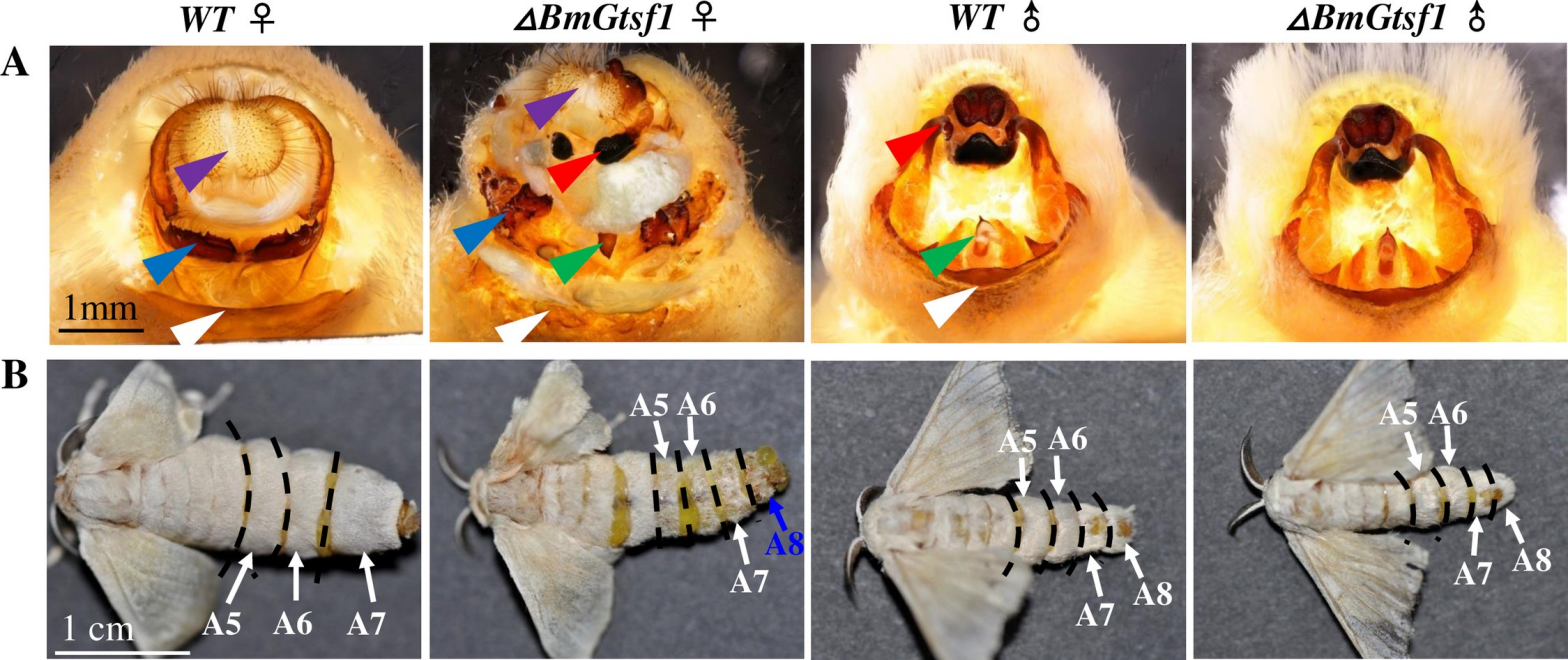
Partial sexual reversal of female *BmGtsf1* mutants. (A) external genitalia of WT and *ΔBmGtsf1* adults. Key to arrows: white, ventral plates; blue, ventral chitin plate; purple, genital papillae; red, clasper (the male specific structure); green, penis (the male specific structure). The red and green arrow showed male specific structures existed in female mutant. (B) Abdominal segment of WT and *BmGtsf1* mutant adults. *BmGtsf1* female mutant developed male specific eight abdominal segments.

Phenotypic analysis of *ΔBmGtsf1* animals revealed a critical role of *BmGtsf1* in female sex determination. Female mutants (the genotype is WZ) displayed intersexual external genitalia with distinct male-specific structures (Fig 2A, red and green arrow). In addition,female mutant adults developed a male-specific eighth abdominal segment (Fig 2B). Male mutants (the genotype is ZZ) developed normal male genitalia and abdominal segmentation (Fig 2B). In *B*. *mori*, female pupae are on average larger and heavier than male pupae. However, both body size and weight of female mutant pupae were reduced and closer to male, while those of male mutant were unaffected (S3 Fig). And we confirmed that the phenotypes were identical among the different individuals.

**Fig 3:**
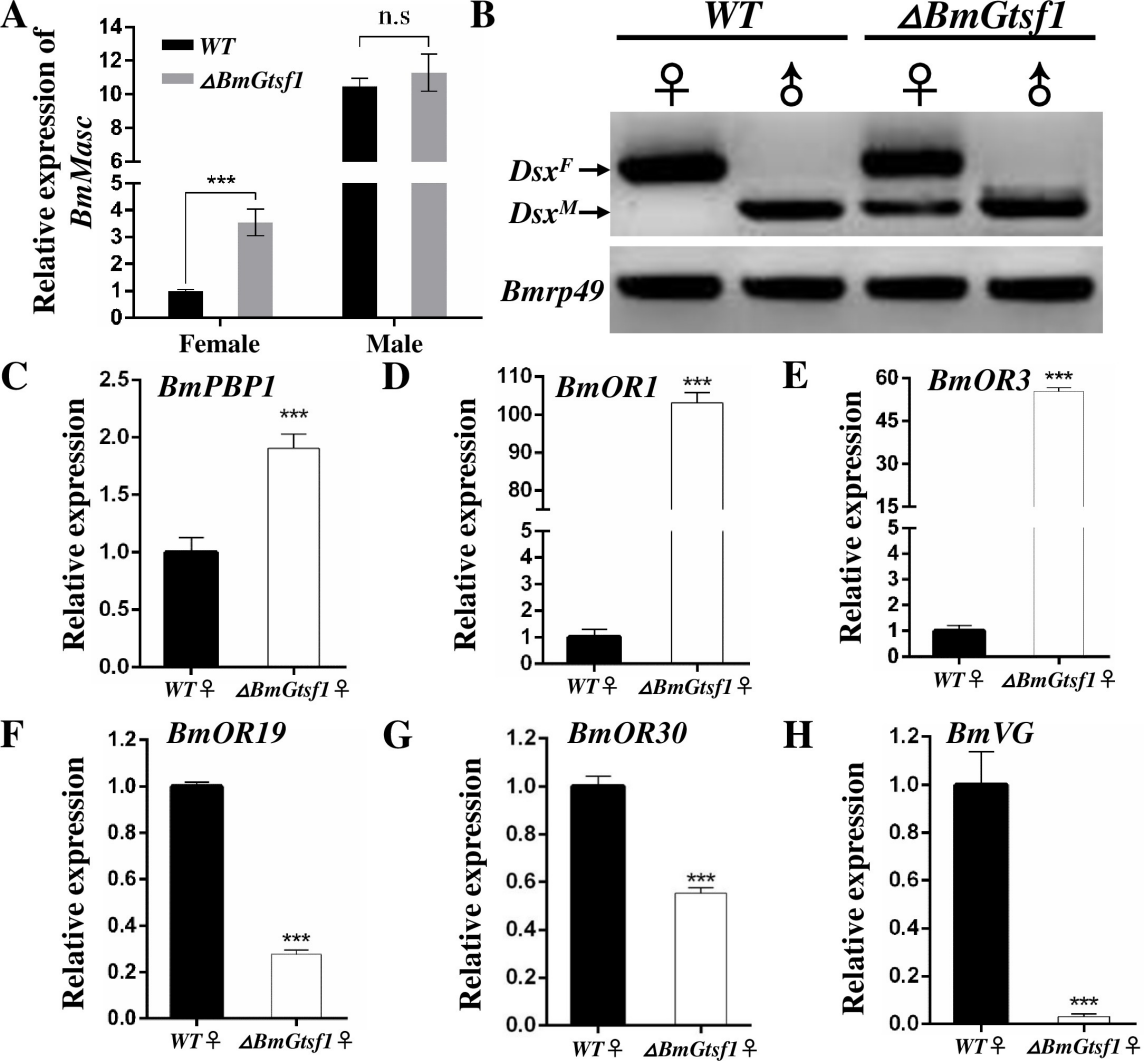
Alternative splicing pattern of *Bmdsx* and qRT-PCR analysis of *BmMasc* and putative target genes of *Bmdsx*. (A) Relative expression of *BmMasc* in gonads of WT and *ΔBmGtsf1* at wandering stage. (B) Splicing patterns of *Bmdsx* in gonads of WT and *BmGtsf1* mutants at wandering stage detected by RT-PCR. (C-H) Relative expression of *BmPBP1* (C), *BmOR1*(D), *BmOR3* (E), *BmOR19* (F), *BmOR30* (G) and *BmVg* (H) in *BmGtsf1* female mutants. RNA extracted from antenna of adults was used for qRT-PCR analyses in Fig 3C–Fig 3G, while the expression of *BmVG* was detected in fat body at wandering stage. Error bars represent ±SD. The asterisks represent statistically significant differences with p < 0.001.

**Fig 4:**
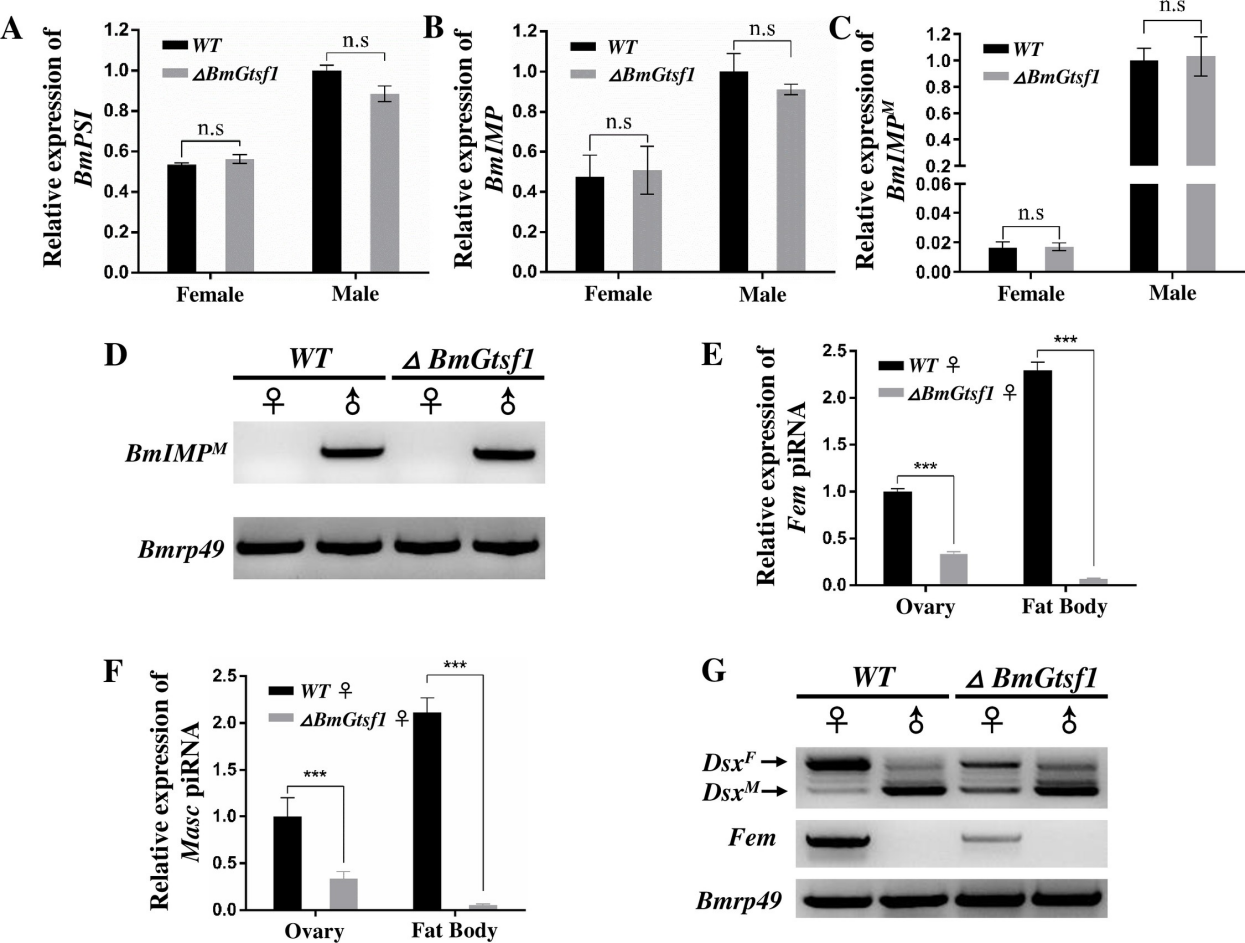
*BmGtsf1* regulate female sex determination via *Fem* piRNA. (A-C) Relative mRNA levels of *BmPSI* (A), *BmIMP* (B) and *BmImp*^*M*^ (C, male-specific *BmIMP*) in gonads of WT and mutants at wandering stage. (D) RT-PCR analysis of *BmImp*^*M*^ in gonads of WT and mutants at wandering stage. (E and F) Relative abundance of *Fem* piRNA (E) and *Masc* piRNA (F) in ovary and fat body of female mutant at wandering stage, the small RNA U6 was used as the internal reference. (G) Splicing patterns of *Bmdsx* and expression of *Fem* precursor in WT and *BmGtsf1* mutant embryoes at 24hpo detected by RT-PCR. The asterisks represent statistically significant differences with p < 0.001. Error bars represent ±SD.

Subsequently, we examined expression levels of *BmMasc*, the upstream regulator of *Bmdsx* [17, 45]. Levels of *BmMasc* transcripts were upregulated by 3.55-fold in the female mutants, but remained unaffected in male mutants (Fig 3A). Disruption of female development of female mutants seems to result from expression of male variants of *Bmdsx*, the conserved downstream effector gene in the *B*. *mori* sex determination pathway [45], as we detected male-specific splicing of *Bmdsx* in female *ΔBmGtsf1* animals (Fig 3B). In addition, we also tested expression of several putative target genes of *Bmdsx*. The mRNA levels of three male-specific expressed genes in olfactory system, *pheromone binding protein 1* (*BmPBP1*), *olfactory receptors 1* (*BmOR1*), *BmOR3*, were significantly increased, while expression of two female olfactory system genes, *BmOR19*, *BmOR30*, and the yolk gene, *vitellogenin* (*BmVg*), were significantly reduced in female mutants (Fig 3C–3H). In contrast, transcripts levels of all these genes were not affected in male mutants (S4 Fig). These results suggested that *BmGtsf1* plays a critical role in the *B*. *mori* female sex determination pathway, particularly through modulating the expression level of *BmMasc*, although more evidences were needed to confirm the direct regulation mechanism. Expression of the auxiliary sex determination factors, *BmIMP* and *BmPSI* [17, 45], appeared not to be affected (Fig 4A–4D). As levels of *Fem* piRNA and *Masc* piRNA were substantially downregulated in female mutants (Fig 4E and 4F), we concluded that *BmGtsf1* mutation primarily affects the sex determination pathway at the level of *Fem* piRNA biogenesis. Through RT-PCR analysis, we found that *BmGtsf1* is involved in initial sex determination pathway at early embryonic stage. In the WT, only faint band for male-specific *Bmdsx* was observed in female. While in the female mutants, male-specific band was enhanced. In addition, the abundance of *Fem* precursor was down-regulated in female mutant (Fig 4G).

To confirm whether the *BmGtsf1* mutation impacts the function of *Fem* piRNA, we constructed four plasmids: *PIZT-Cas9-EGFPsgRNA* (control), *PIZT-Cas9-U6-Fem* (to overexpress *Fem* piRNA, *Fem-OE*), *PIZT-Cas9-Gtsf1sgRNAs* (to knock out *Gtsf1*, *Gtsf1-KO*), *PIZT-Cas9-Gtsf1sgRNAs-U6-Fem* (to knock out *Gtsf1* and overexpress *Fem* piRNA, *Gtsf1-ko-Fem-OE*) to perform a rescue assay in BmN cells (S5A Fig). BmN cells transfects with *PIZT-Cas9-EGFPsgRNA* was served as control. In accordance with the results *in vivo*, *BmGtsf1* depletion resulted in *Fem* piRNA down-regualtion and *BmMasc* up-regulation in BmN cells. We found that the abundance of *Fem* piRNA can be rescued through U6 promotor overexpressing. However, the up-regulation of *BmMasc* caused by *BmGtsf1* depletion cannot be rescued, although the level of *Fem* piRNA was much higher than control (S5B Fig and S5C Fig). These data suggested that *BmGtsf1* is also required for the function of *Fem* piRNA. We next overexpressed *BmGtsf1 in vivo* (*BmGtsf1-OE*) using *IE1* promoter (S6A Fig), and the results showed that neither the expression of *BmMasc* nor the splicing of *Bmdsx* was altered (S6B Fig and S6C Fig). No detectable phenotype was observed in *BmGtsf1* overexpressed animals, and all the adults were fertile, indicating *BmGtsf1* does not has dosage effect (S6D Fig). In summary, from our data we concluded that *BmGtsf1* controls female sex determination through assisting the production and function of *Fem* piRNAs, which are needed to effectively repress the male determining gene *BmMasc*.

### *BmGtsf1* mutation results in gametogenesis defects in both male and female

**Fig 5:**
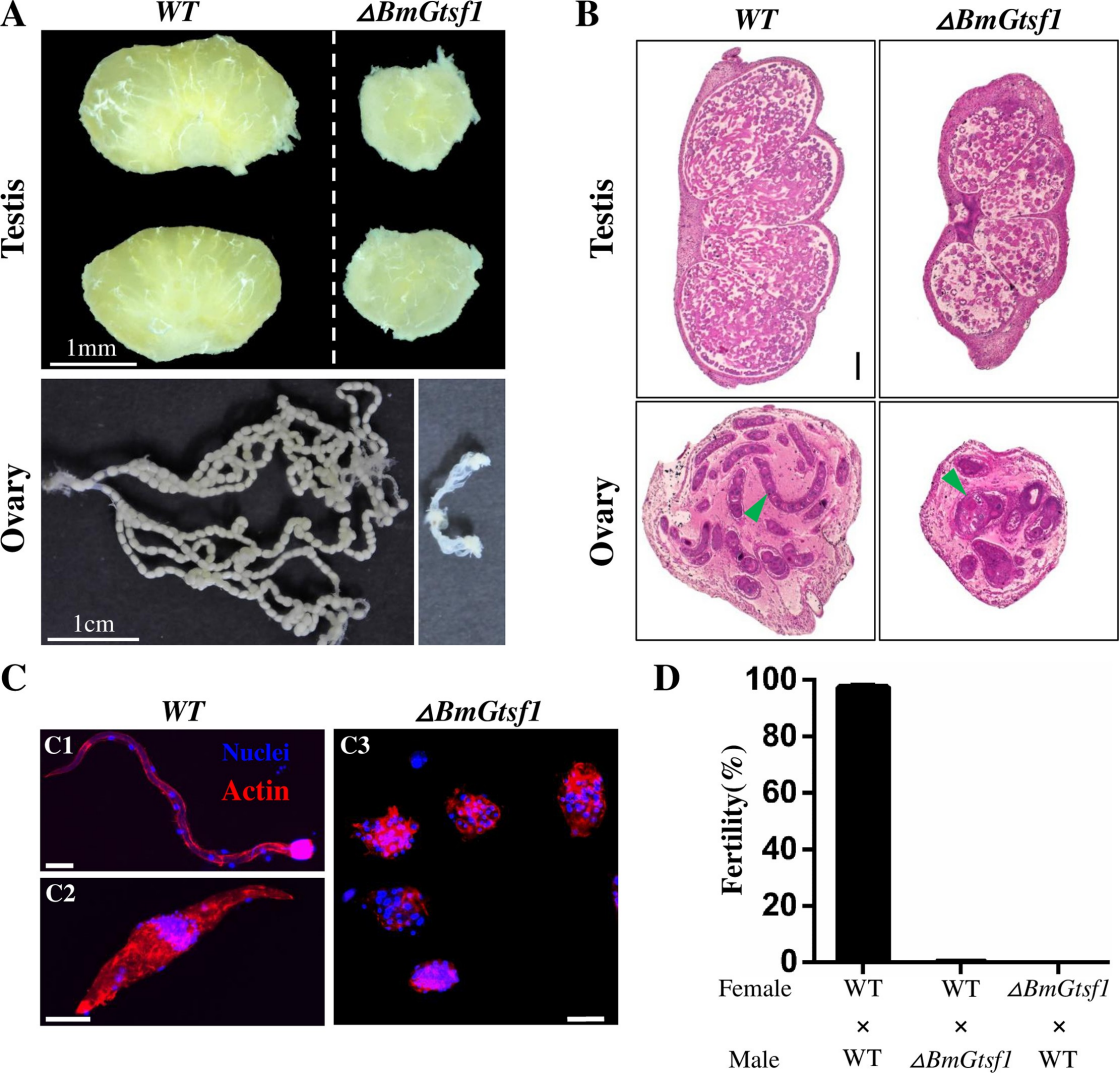
*BmGtsf1* mutation results in gametogenesis defects. (A) Morphological analysis of gonads in the WT and mutant adults. (B) Internal structure of gonads in the WT and mutant animals at wandering stage using hematoxylin and eosin staining. Scale bar: 200 μm. The green arrow indicates ovariole. (C) Sperm bundles from WT and mutant male adults. C1, eupyrene sperm bundles; C2, apyrene sperm bundles; C3, abnormal sperm bundles in *BmGtsf1* mutant. (D) Fertility of WT and *BmGtsf1* mutants, n = 20.

To investigate the effects of *BmGtsf1* mutation on gonadal development, we performed a morphological examination. The testes of the adult male mutants were highly atrophied (Fig 5A, top row), nevertheless the weights of male mutants were unchanged (S3B Fig). Wild-type females contain two ovaries, each of which consists of four ovarioles filled with eggs at various stages of development. In contrast, the ovarioles of female mutants were severely degraded, neither obvious ovariole nor eggs were observed (Fig 5A, bottom row). To further examine the internal structures of the female and male gonads, we stained paraffin-embedded tissue sections with hematoxylin and eosin at the wandering stage. This examination also revealed that the testes development of male mutants were affected. They were much smaller in overall size and the sperm count was greatly reduced (Fig 5B, top row). In *B*. *mori*, there are dimorphic sperm: eupyrene sperm bundles and apyrene sperm bundles [46]. In the WT adult males, both kinds of sperm bundles were matured, by comparison, spermatogenesis was arrested in male mutant, and we can only found round spermatogonium (Fig 5C). Similarly, the ovary of mutant was also atrophied, and no clear ovariole was observed (Fig 5B, bottom row). As a result of defective gametogenesis, both male and female mutants were sterile (Fig 5D). Taken together, these data demonstrated that *BmGtsf1* deficiency will lead to gametogenesis defects in both male and female silkworms.

### BmGTSF1 interacts with BmSIWI

**Fig 6:**
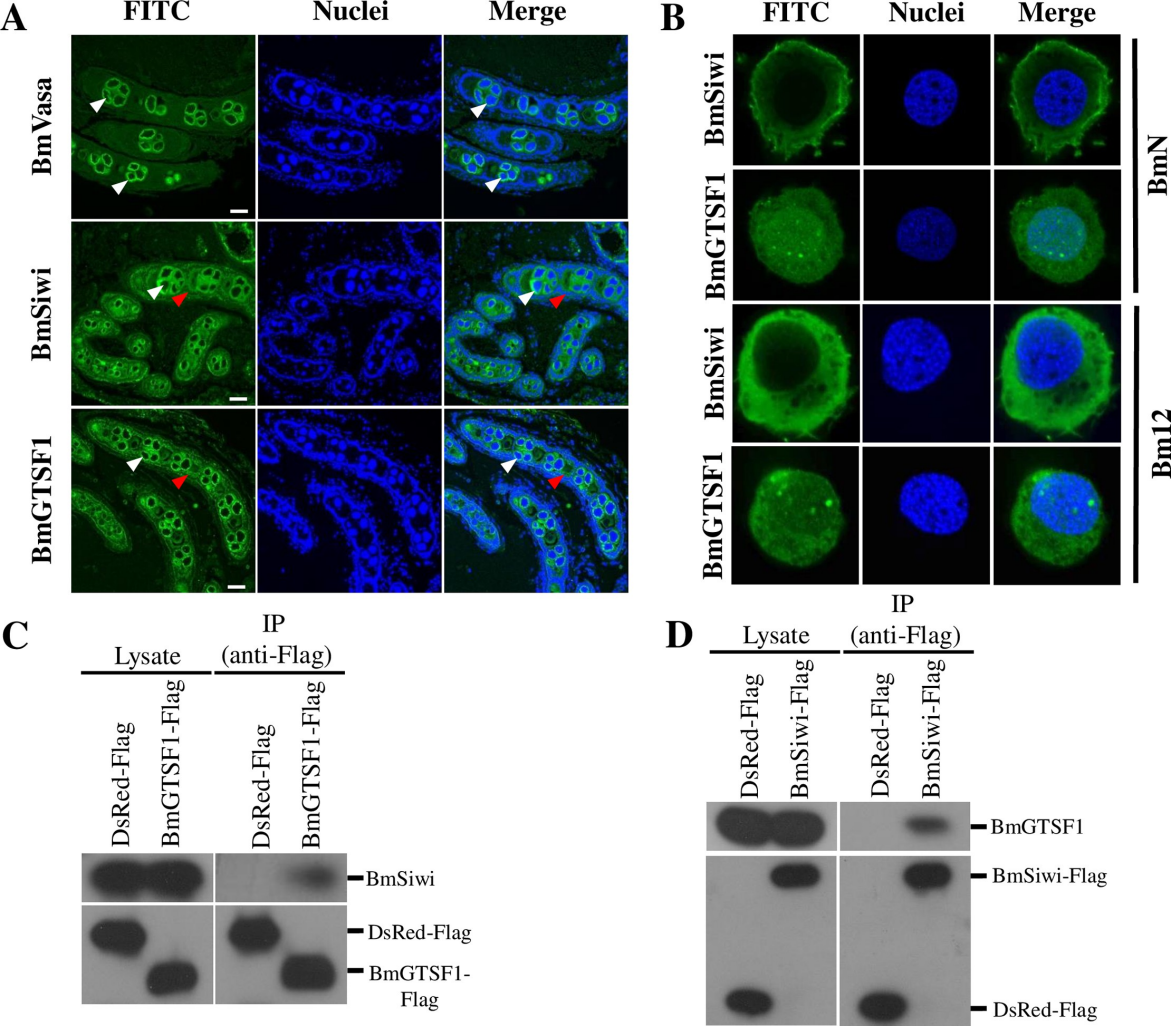
BmGTSF1 interacts with BmSIWI. (A) Localization of BmVasa, BmSIWI and BmGTSF1 in the ovaries of *B*. *mori*. A FITC-conjugated secondary antibody was used for fluorescence detection and Hoechst staining (blue) showed the locations of the nuclei. Scale bar, 50μm. The white and red arrow indicates germ line cells and somatic cells respectively. (B) Intracellular localization of BmSIWI and BmGTSF1 in BmN cells and Bm12 cells. (C) Immunoprecipitation followed by western blot showed that endogenous BmSIWI immunoprecipitated with BmGTSF1-Flag rather than DsRed-Flag. (D) Immunoprecipitation followed by western blot showed that endogenous BmGTSF1 immunoprecipitated with BmSIWI-Flag rather than DsRed-Flag.

Given the fact that GTSF1 interacts with PIWI in both mouse and *Drosophila* [20–22], we next examined the relationship of the two proteins in the silkworm. Firstly, we detected their localization in the gonads. For clear analysis of immunostaining results, we used a primary antibody recognizing BmVasa, which is a germline specific marker in insects, to differentiate germline cells and somatic cells. In ovary, BmGTSF1 and BmSIWI were present in both germline and surrounding somatic cells (Fig 6A). In testis, the distribution of BmVasa, BmGTSF1 and BmSIWI was almost overlapping, and restricted to spermatogonium (S7 Fig). We also examined the cellular localization of BmSIWI and BmGTSF1 in BmN and Bm12 cells, which were derived from *B*. *mori* ovary. The immunostaining results showed that BmSIWI located to the cytoplasm, while BmGTSF1 localized in both nucleus and cytoplasm (Fig 6B), indicating these two proteins have different intracellular localization. To test for a direct interaction between BmGTSF1 and BmSIWI, we fused BmGTSF1 (BmGTSF1-Flag) and BmSiwi (BmSiwi-Flag) C-terminally with a Flag tag and used a monoclonal anti-Flag antibody for immunoprecipitation in BmN cells. DsRed fused C-terminally with Flag tag (DsRed-Flag) was served as control. The western blot followed immunoprecipitation confirmed that the BmGTSF1-Flag coprecipitated with endogenous BmSIWI (Fig 6C), more importantly, the reciprocal immunoprecipitation experiment showed that BmSIWI-Flag also coprecipitated with endogenous BmGTSF1 (Fig 6D). In conclusion, BmGTSF1 and BmSIWI showed the same localization in the gonads of *B*.*mori*, and they physically interacted.

### *BmGtsf1* is essential for piRNA induced transposon silencing

**Fig 7:**
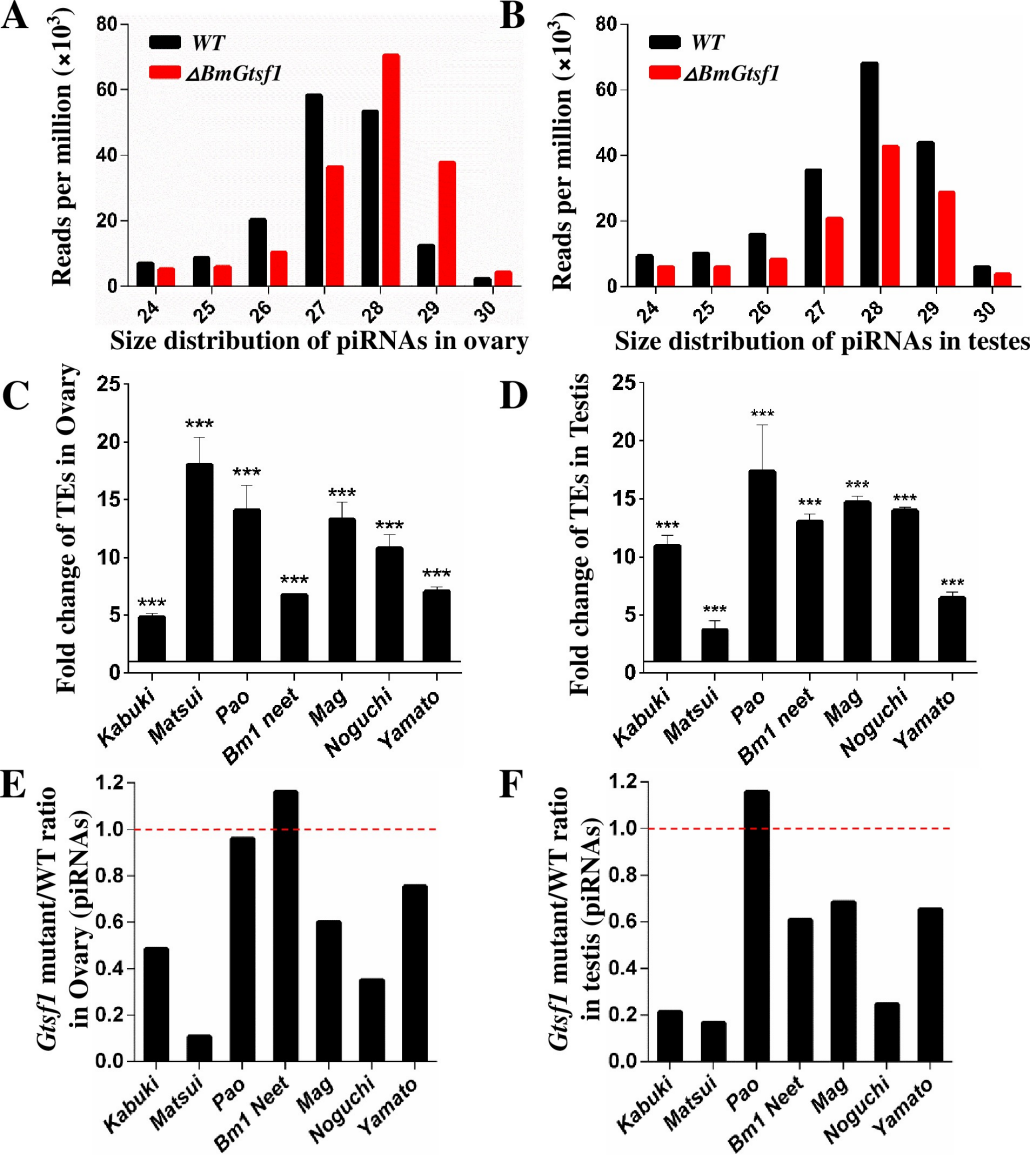
*BmGtsf1* mutation mediated piRNA pathway dysregulation. (A and B) Size distribution of piRNAs in ovary (A) and testis (B) of WT and *BmGtsf1* mutant. (C and D) Fold change of transposons in ovary (C) and testis (D) of *BmGtsf1* mutant compared to WT. (E and F) Relative abundance of piRNAs correspond to detected transposons.

Given the well-known functions of *Gtsf1* in *Drosophila* and mouse, we next tested whether *BmGtsf1* is also required for piRNA pathway in *B*.*mori*. To this end, we analyzed piRNA abundance and transposons expression level in the gonads of WT and mutant by transcriptome analysis and qRT-PCR. In ovaries of *ΔBmGtsf1* animals, abundance of 24–27nt long piRNAs was greatly decreased, while the amount of 28–30nt long piRNAs was slightly increased compared to wild-type ovaries (Fig 7A). Moreover, the abundance of 24–30nt long piRNAs was remarkably reduced in testis suggesting that *BmGtsf1* is required for piRNA maturation in the gonads (Fig 7B).

In addition, we found that lack of *BmGtsf1* activity resulted in derepression of numerous transposons in the gonads of mutant animals (S8A Fig and S8B Fig). More specifically, we examined mRNA levels of seven well annotated transposons [48, 49] by qRT-PCR. The expression of all of these transposons were greatly upregulated (Fig 7C and 7D). In line with the observation, abundance of piRNAs correspond to these transposons were declined (Fig 7E and 7F), except for the piRNAs correspond to *Pao* and *Bm1-Neet* (*Bm1-related_Neet*). In addition, levels of *Fem* piRNA was severely reduced in ovaries of *ΔBmGtsf1* animals (Fig 4E). *Fem* piRNA depletion was also observed in *BmSiwi* mutant animals [18], supporting the notion that BmGTSF1 interacts with BmSIWI. Altogether, our data demonstrated that *BmGtsf1* is involved in the piRNA pathway. These results also revealed that *BmGtsf1* regulate female sex determination via piRNA pathway.

## Discussion

The functions of GTSF1 have thus far been examined in three species, *Drosophila*, mouse and *C*. *elegans* [20–22, 50]. Despite the fact that GTSF1 is conserved, its functions appear to have diversified in these three species. In both *Drosophila* and mouse, GTSF1 is necessary for piRNA pathway, however, the mechanisms appear to be quite different. In *Drosophila*, DmGTSF1 localizes to the nucleus, and is required for Piwi-piRISC-mediated transcriptional transposon silencing but not for piRNA biogenesis [20, 21]. While in mouse, GTSF1 localizes to both cytoplasm and nucleus, and is essential for secondary but not for primary piRNA biogenesis [22]. In *C*. *elegans*, GTSF1 does not participate in the piRNA pathway, but instead is involved in the assembly of ERI (enhanced RNAi) complex [50]. In *B*. *mori*, as *BmGtsf1* mutations caused derepression of TEs and dysregulation of piRNAs, we concluded that BmGTSF1 is not only necessary for transposon silencing but also for piRNA biogenesis. In addition, our co-immunoprecipitation experiments suggested that BmGTSF1 interacts with BmSIWI, such an interaction was also observed in *Drosophila* and mouse [20–22], suggesting a relatively conserved function of GTSF1 in piRNA pathway. However, although these proteins were interacted, their cellular localization was different. We speculated that BmGTSF1 may have another role in nucleus, for example, it might participate in transporting piRNA precursors. There are two facts in accordance with our hypothesis. The one is that BmGTSF1 contains two CHHC-type Zinc finger motifs (S2A Fig), the predicted RNA binding domain. The other is that we observed condensation of BmGTSF1 in nuclues (Fig 6B). The roles of GTSF1 in *B*. *mori* and *Drosophila* seemed quite different, we supposed the different mechanism is related to the fact that transcriptional transposon silencing is absent in the silkworm [51]. In *Drosophila* and mouse, *Gtsf1* is predominantly expressed in germ cells. While in the silkworm, *BmGtsf1* was expressed in practically all tissues, consistent with the fact that BmGTSF1 is not only involved in the germline piRNA pathway but also in somatic sex determination.

piRNAs derived from *Fem* precursor serve as the primary signal for *B*. *mori* female sex determination by repressing the male determining gene *Masc*. Nevertheless, not all of the genes involved in processing and maturation of piRNAs participate in *Masc* repression. To date, only the core components of the piRNA pathway, *BmSiwi*, and its cofactor *BmAsh2*, have been shown to be involved in *B*. *mori* sex determination. In contrast, the factors *BmAgo3* and *BmMael* appear not to be required for sex determination [18, 44]. This present study includes another piRNA pathway factor *BmGtsf1* in the regulation of female sex determination in *B*. *mori*. Compromising the activity of *BmGtsf1* results in partial sex reversal of WZ individuals, while no observable effects on sex determination were detected in ZZ male individuals. In accordance with a role in piRNA mediated repression of *BmMasc*, expression levels of *BmMasc* were significantly upregulated in WZ mutant individuals. As levels of two auxiliary sex determination factors *IMP* and *PSI* were unaffected, this failure to repress *BmMasc* can be likely attributed to a lack of *Fem* piRNA activity. Consistent with this interpretation, the abundance of *Fem* piRNA was found to be significantly low in WZ mutant individuals. Concomitantly, levels of *Masc* piRNAs derived from *BmMasc* mRNA through the ping-pong cycle were also drastically reduced. Furthermore, overexpression of *Fem* piRNAs in *BmGtsf1* depletion BmN cells cannot restore repression of *BmMasc*. Thus, we concluded that *BmGtsf1* is involved in female sex determination by assisting the proper processing of functional *Fem* piRNAs to repress *BmMasc*. However, we proposed that *BmGtsf1* might be an auxiliary factor for female sex determination, since *BmGtsf1* mutation caused partial sex reversal of female animals.

piRNA pathway was previously reported to act primarily as a silencing system which prevents TE expression in animal gonads [26, 31, 21, 52]. However, in the silkworm, piRNA pathway is not only required for TE silencing but, as shown in this study, is also involved sex determination. Hence, there seemed to be a somatic piRNA pathway which specifically works in the silkworm sex determination pathway. Consistent with our speculation, we detected that expression of several transposons were barely changed in mutant fat body (S8C Fig and S8D Fig), by contrast, the *Fem* and *Masc* piRNA level were significantly downregulated (Fig 4E and 4F). We assumed that *BmSiwi* was the core component of this somatic piRNA pathway, because BmAsh2 and BmGTSF1, the only two known piRNA factors required for *B*. *mori* sex determination, all interact with BmSIWI. Depletion of BmGTSF1; a factor interacting with BmSIWI, showed similar phenotypes as *BmSiwi* mutants does [18]. Nevertheless, the sex reversal phenotypes were more distinct in *BmGtsf1* mutants. We speculated that the more distinct phenotypes were attributed to the extra function of BmGTSF1 in nucleus.

As the piRNA pathway is responsible for genome stability in animal gonads, depletion of piRNA factors often results in sterility attributed to DNA damage caused by TEs derepression [52–54]. *GTSF1* mutations lead to severely deformed ovaries and female sterility in *Drosophila*, while the testes appear unaffected [20, 21]. Mouse *GTSF1* mutation resulted in much smaller testis and spermatogenesis defects, by contrast, no abnormalities were detected in female [22]. Namely, *GTSF1* mutation specifically affects gonadogenesis of the single sex in *Drosophila* and mouse. Different from the sex-specific role in mouse and *Drosophila* germ line, we found that *BmGtsf1* mutation would result in gametogenesis and gonadogenesis defects in both males and females. We showed that, similar to BmSIWI, BmGTSF1 is required for proper differentiation and maturation of eggs. We assumed that ovarian phenotypes can be attributed to dysregulation of both piRNA pathway and the sex determination pathway. In line with this, the expression of *BmVG*, a gene which predominantly expressed in female fat body, was drastically down-regulated in the female mutant individuals. An interesting finding was that *BmGtsf1* is also required for spermatogenesis. The phenotype appeared to be caused by TEs derepression only, since *BmGtsf1* mutation had few effects on male development and sex determination. We concluded that *BmGtsf1* is essential for *B*. *mori* TE silencing during gametogenesis, although more direct evidence is needed to prove a causal relationship between TEs derepression and defects in gametogenesis.

**Fig 8:**
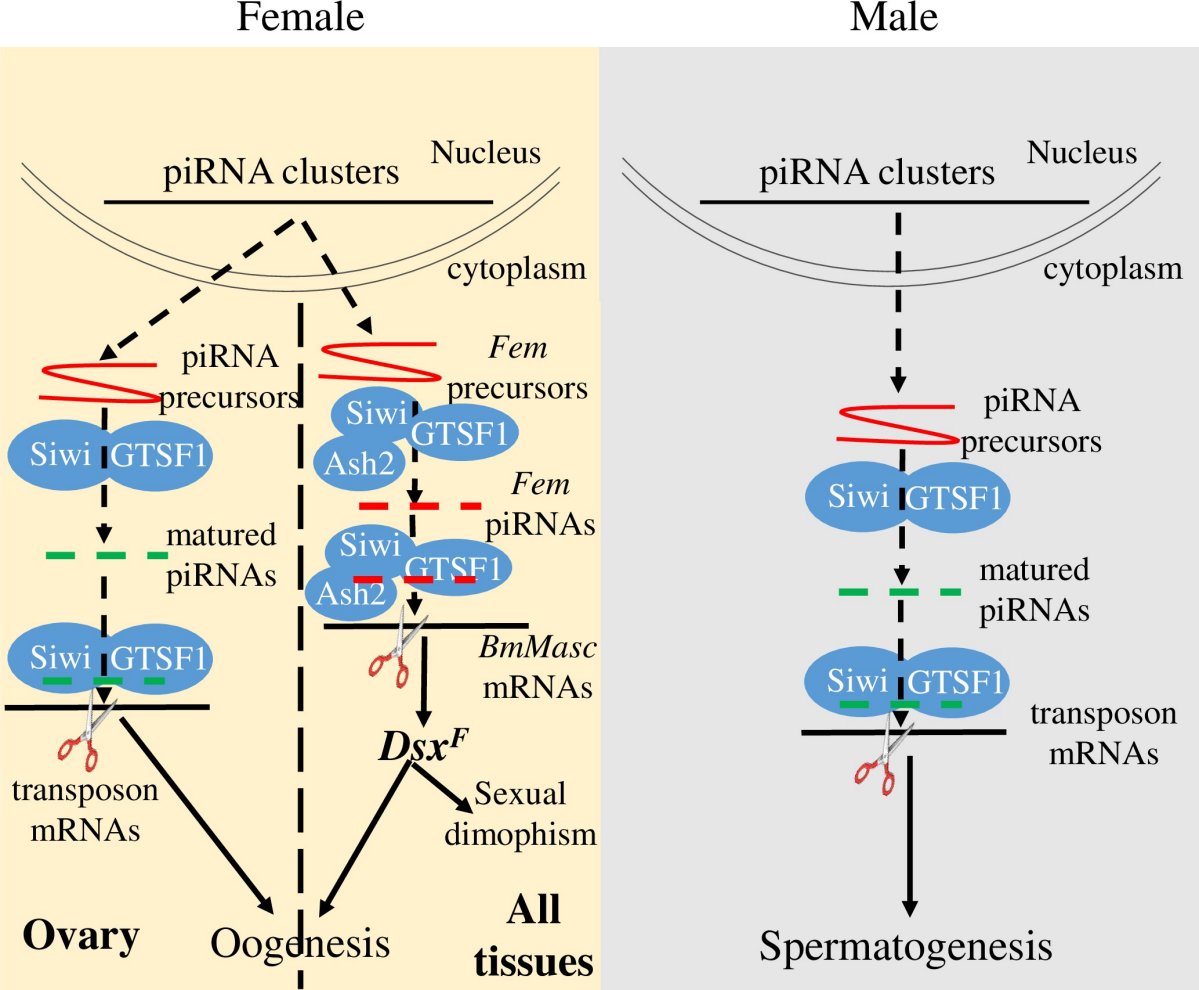
Proposed model for functions of BmGTSF1 in *B*. *mori*. Although BmGTSF1 interacts with BmSiwi, the function of BmGTSF1 is different in male and female. In female, BmGTSF1 is reqiured not only in canonical TE silencing but also sex determination, both of which are vital for oogenesis. It was reported that another factor BmAsh2, which interactes with BmSiwi, is also involved in sex determination, although whether BmAsh2 interactes with BmGTSF1 is unkonwn. While in male, BmGTSF1 is only essential for transposon silencing, which ensures proper spermatogenesis.

In conclusion, our data provided a first functional analysis of BmGTSF1, the *Drosophila* ortholog of GTSF1 in the silkworm. We showed that BmGTSF1 is a new addition to the piRNA processing factors required not only in canonical TE silencing but also female sex determination pathway in *B*. *mori* (Fig 8). Although the piRNA pathway is involved in both TE silencing and female sex determination in the silkworm, not all of the factors regulating piRNA activity participate in both pathways. For future studies, it will be necessary to identify new piRNA factors and test whether or not they are also required in *B*. *mori* sex determination. Such studies will contribute to a better understanding and distinction of these two vitally important roles of the piRNA pathway in silkworm development.

## Materials and methods

### Silkworm strain and cell line

The multivoltine, nondiapausing silkworm strain, Nistari, was used in this study. The larvae were reared on fresh mulberry leaves under standard conditions [55]. The cell line BmN and Bm12, which are derived from silkworm ovary, were used for transfection and immunofluorescent staining. The cell line was cultured at 27°C in TC100 insect medium with 10% fetal calf serum (FBS) [56].

### Information of *BmGtsf1* gene

*BmGtsf1* is located on chromosome11 according to SilkBase (http://silkbase.ab.a.u-tokyo.ac.jp/cgi-bin/index.cgi), and the gene ID of *BmGtsf1* in NCBI (https://www.ncbi.nlm.nih.gov/gene/101745848) is 101745848.

### Analysis of mutants genotype

The genomic DNA of the mutants was using standard SDS lysis-phenol treatment, incubated with proteinase K, treated with RNase treatment, and purified. 150ng of the genomic DNA was used as template, and the primer pair, *Fem-seq-F* and *Fem-seq-R* (S1 Table), which flank *Fem* precursor was used (S1 Fig). To confirm the genotype of early embryo, the genome was extracted according to the manufacturer's instructions for TRIzol reagent.

### RNA isolation and cDNA synthesis

Total RNA was isolated from the tissues of three individual mutants or WT animals using the TRIzol reagent according to the manufacturer's instructions, followed by treatment with DNase I and purification. The quality of total RNA was confirmed by agarose gel electrophoresis. An aliquot of 1 μg of the total RNA was used to synthesize cDNA using ReverAid First Strand cDNA Synthesis Kit. For piRNA detecting, the total RNA was firstly incubated with piRNA specific stem-loop primer at 65°C for 5min, followed by normal cDNA synthesis steps according to the manufacturer's instructions.

### Quantitative real-time PCR (qRT-PCR)

qRT-PCR analyses were performed using SYBR Green Real-time PCR Master Mix to analyze mRNA levels of selected genes and piRNAs abundance. mRNA levels were normalized to levels of *B*. *mori ribosomal protein 49* (*Bmrp49*). The relative expression levels of piRNAs were measured by using the stem-loop method [57, 58], and the small RNA U6 was used as the internal reference. Three independent biological replicates of each qRT-PCR analysis were performed. All the primers used for qRT-PCR were listed in S1Table.

### Western blot analysis

For western blot analysis, the following primary antibodies were used: polyclonal rabbit anti-BmGTSF1 (1:5000), polyclonal rabbit anti-BmSIWI (1:2000), monoclonal mouse anti-β-actin (1:5000), monoclonal mouse anti-Flag (1:5000). The peptides corresponding to the full length of BmGTSF1 was synthesized and used for generating polyclonal rabbit anti-BmGTSF1 primary antibody. For the secondary antibody, horseradish peroxidase-conjugated goat anti-rabbit and anti-mouse IgG (1:5000) were used. The ECL Plus Western blotting Detection Kit was used to detect the protein signal.

### Paraffin sectioning and hematoxylin-eosin staining

Gonads of mutants and WT animals were dissected from the wandering stage larvae and prefixed with Qurnah's fixative (anhydrous ethanol: acetic acid: chloroform, 6:1:3 (v/v/v)) for 24h. Samples were dehydrated using anhydrous ethanol for three times (1h per time), followed by clearing for three times (10min per time) using xylene. Tissues were embedded in paraffin overnight, and were cut into cross-sections (5 μm) with a Leica RM2235 microtome. The sections were rehydrated and then stained using a mixture of hematoxylin and eosin to visualize morphology. The stained sections were photographed by using an Olympus BX53 microscope.

### Gonads immunostaining

The gonads paraffin-embedded sections were firstly rehydrated by steps as follow: xylene for twice, 10min per time, 95% ethanol for twice, 5min per time, 80% ethanol for 5min, 70% ethanol for 5min, H_2_O for twice, 3min per time, followed by PBS (phosphate buffered saline) for 5min. Sections were treated with 0.1% trisodium citrate containing 0.1% Triton X-100 for 10min at room temperature for antigen retrieval. The samples were washed using PBS for twice, followed by blocking with 1% BSA (bovine serum albumin) for 1 hour at room temperature. The sections were then incubated with primary antibodies for 48 hours at 4°C. Primary antibodies used for immunostaining were as follow: polyclonal rabbit anti-BmGTSF1 (1:500), polyclonal rabbit anti-BmSIWI (1:200), polyclonal rabbit anti-BmVasa (1:200). The samples were washed with PBS for three times and incubated with FITC-AffiniPure Goat Anti-Rabbit IgG (H+L) (1:200) secondary antibody for 2 hours at room temperature. The samples were washed for once and treated with Hoechst33258 for 10min at room temperature. The samples were washed for four times and analyzed with an Olympus BX53 microscope.

### Immunostaining of BmN and Bm12 cells

BmN and Bm12 cells were seed on cover glass put in culture dish. 12 hours after cell passaging, cells were fixed (Immunol Staining Fix Solution) for 1 hour at room temperature. The samples were washed twice for 5min and then permeabilized with 0.1% Triton X-100 in 0.1% trisodium citrate for15 minutes. After washing with PBS for 5min, the samples were blocked using 1% BSA for 2 hours at room temperature. The cells were incubated with primary antibodies for 48 hours at 4°C. Following three washing steps, cells were incubated with secondary antibody for 2 hours at room temperature. The antibodies used in cell lines staining were the same as gonads immunostaining. The samples were washed for once and treated with Hoechst33258 for 10min at room temperature. After washing for 4 times for 10min, the results were analyzed using Olympus Fluoview FV1000 Confocal Microscope.

### Immunoprecipitation assay

Coding sequences of BmSIWI, BmGTSF1 and DsRed tagged with 3×FLAG were cloned into pIZT/V5-His plasmid. The plasmids were transfected into the BmN cell line respectively using Effectene Transfection Reagent according to the manufacturer's instructions. Crude proteins from one 60mm dish cells were collected using lysis buffer (50 mM Tris HCl, pH 7.4, 150 mM NaCl, 1 mM EDTA, 1% TRITON X-100) 72 hours after transfection, and then used for immunoprecipitation. Immunoprecipitation was performed using ANTI-FLAG M2 Affinity Gel. Immunoprecipitates were washed ten times with TBS (Tris Buffered Saline, 50 mM Tris HCl,150 mM NaCl, pH 7.4) at 4°C, 5min per time. The immunoprecipitation results were detected using western blot.

### CRISPR/Cas9 system mediated mutant construction

A binary transgenic CRISPR/Cas9 system was applied to construct *BmGtsf1* mutant. The plasmids construction and the silkworm germline transformation were described previously [44, 45]. Briefly, two silkworm lines were constructed, one line expressed Cas9 nuclease under the control of the *B*. *mori nanos* (*nos*) promoter, the other line expressed two guide RNAs activated by U6 promoter [44, 45]. The mixture of transformation plasmids and helper plasmids were microinjected into preblastoderm G0 embryos for germline transformation.

### Mutagenesis analysis

Genomic DNA was extracted from the somatic mutants using standard SDS lysis-phenol treatment, incubated with proteinase K, treated with RNase treatment, and purified. To perform gene-specific PCR amplification, 100 ng of the genomic DNA was used as template, and the primer pair, *BmGtsf1-seq-F* and *BmGtsf1-seq-R* (S1 Table), which flank both targets was used. The PCR products were extracted and cloned into the pJET-1.2 vector, and 20 clones from 5 individual were sequenced. The results of sequencing are listed in S2 Table.

### RNA-seq analysis

Total RNA from the testes and ovaries of wandering stage animals was extracted from three individual animals of *ΔBmGtsf1* and WT, and mixed together. The total RNA was used for both mRNA sequencing and small RNA sequencing analysis. For mRNA sequencing, the total RNA was firstly enriched and then fragmented, and used for cDNA synthesis and library construction. The library was sequenced using BGISEQ-500 technology, raw data was qualified, filtered, and mapped (SOAPnuke, HISAT, Bowtie2) to the reference silkworm genome database (http://sgp.dna.affrc.go.jp/KAIKObase/), mRNA abundance was measured by fragment per kilobase of exon per million fragments mapped (FPKM). The data was then mapped to 1,811 silkworm transposons [59, 60] and analyzed the transposon expression. For small RNA sequencing, RNAs ranging in size between 18 and 32 nucleotides (nt) were gel-purified and used for library construction and sequencing (BGISEQ-500 technology). The generated reads were filtered and mapped to the silkworm genome (http://sgp.dna.affrc.go.jp/KAIKObase/). The mapped reads from 24 to 30nt in length were further mapped to 1811 silkworm transposons [59, 60] allowing no mismatches. These perfect mapping reads were defined as piRNAs. The relative abundance of piRNAs were measured by reads per million (RPM) by normalizing to the total number of perfect genome mapped sRNAs in each library [61].

### Fluorescent staining of sperm bundles

Testes of mutant and WT animals were dissected from adult animals. Next, the testes were avulsed and the contents were fixed for 1 hour at room temperature. Samples were then washed three times using PBS, followed by staining with TRITC Phalloidin for 1 hour. Nuclei were stained with Hoechst33258 for 10 min at room temperature. Samples were washed three times with PBS, dropped onto a microslide, and immediately analyzed by a fluorescence microscopy (Olympus, BX53).

### Statistics

Experimental data were analyzed with the Student's t-test. The asterisks indicate different *p* value, *, p<0.05; **, p<0.01; ***, p<0.001.

## Supporting information

S1 Table Primers used in this work. (XLSX)

S2 Table Summary of the mutagenesis analysis. (XLSX)

S1 Fig Analysis of mutants’ genotype. The primer pair flank *Fem* precursor was used to confirm genotype of mutants and embryos.
(TIF)

S2 Fig CRISPR/Csa9 system mediated *BmGtsf1* mutant construction. (A) Schematic diagram of the *BmGtsf1* gene structure and sgRNA-target sites. The two sgRNA target sites located on the sense strand in exon 3. (B) Diverse types of mutations at *BmGtsf1* locus detected by sequencing. The A labelled in light blue means nucleotide replacement. (C and D) The depletion efficiency confirmed by qRT-PCR (C) and western blot (D).
(TIF)

S3 Fig Body size of WT and mutant pupa. (A) *BmGtsf1* female mutant showed smaller body size compared with that of WT. (B) Pupa weight of WT and *BmGtsf1* mutants. The red bar is average value, n = 30. The asterisks (***) means *p* value < 0.001, and n.s means there is no significant difference between the groups.
(TIF)

S4 Fig qRT-PCR analysis of male or female specific genes in male mutants. (A-F) Relative expression of *BmPBP1* (A), *BmOR1*(B), *BmOR3* (C), *BmOR19* (D), *BmOR30* (E) and *BmVg* (F) in *BmGtsf1* male mutants. RNA extracted from antenna of adults was used for qRT-PCR analyses in Fig 3C–Fig 3G, while the expression of *BmVG* was detected in fat body at wandering stage. Error bars are ± SD.
(TIF)

S5 Fig *BmGtsf1* is essential for the function of *Fem* piRNA. (A) Schematics of the plasmids used in the rescue assay. (B and C) Relative abundance of piRNA abundance (B) and mRNA levels (C) in BmN cells transfected with different plasmids. Error bars are ±SD. The asterisks represent significant differences with p < 0.05.
(TIF)

S6 Fig No obvious phenotype was detected in *BmGtsf1* overexpression lines. (A and B) Relative expression of *BmGtsf1* (A) and *BmMasc* (B) in overexpression lines. (C) Splicing patterns of *Bmdsx* in WT and *BmGtsf1* overexpression line detected by RT-PCR. (D) Fertility of *BmGtsf1* overexpression line, n = 20. RNA extracted from gonads was used for RT-PCR and qRT-PCR analyses.
(TIF)

S7 Fig Immunostaining of BmVasa, BmSIWI and BmGTSF1 in *B*. *mori* testis. (A-C) Localization of BmVasa (A), BmSIWI (B) and BmGTSF1 (C) in the testes of *B*. *mori*. A FITC-conjugated secondary antibody was used for fluorescence detection and Hoechst staining (blue) showed the locations of the nuclei. Scale bar, 50 μm.
(TIF)

S8 Fig RNA-seq analysis reveals up-regulated transposons in gonads and qRT-PCR analysis reveals identical transposon levels in fat body. (A and B) RNA-seq analysis of relative transposon levels in ovary (A) and testis (B) of WT and *BmGtsf1* mutant. (C and D) Relative mRNA levels of transposon in fat body of female and male mutants.
(TIF)
