# Correction: *Gtsf1* is essential for proper female sex determination and transposon silencing in the silkworm, *Bombyx mori*

**DOI:** 10.1371/journal.pgen.1009572

**Published:** 2021-05-17

**Authors:** Kai Chen, Ye Yu, Dehong Yang, Xu Yang, Linmeng Tang, Yujia Liu, Xingyu Luo, James R. Walters, Zulian Liu, Jun Xu, Yongping Huang

The eighth author’s name is spelled incorrectly. The correct name is: James R. Walters. There is also an error in affiliation 3 for author James R. Walters. The correct affiliation 3 is: Department of Ecology and Evolutionary Biology, University of Kansas, KS, United States of America.
