# Supplementary figures and images for: Gtsf1 is essential for proper female sex determination and transposon silencing in the silkworm, Bombyx mori
Source: PLoS Genet. 2020 Nov 2;16(11):e1009194. doi: 10.1371/journal.pgen.1009194 (PMC7660909; doi:10.1371/journal.pgen.1009194)

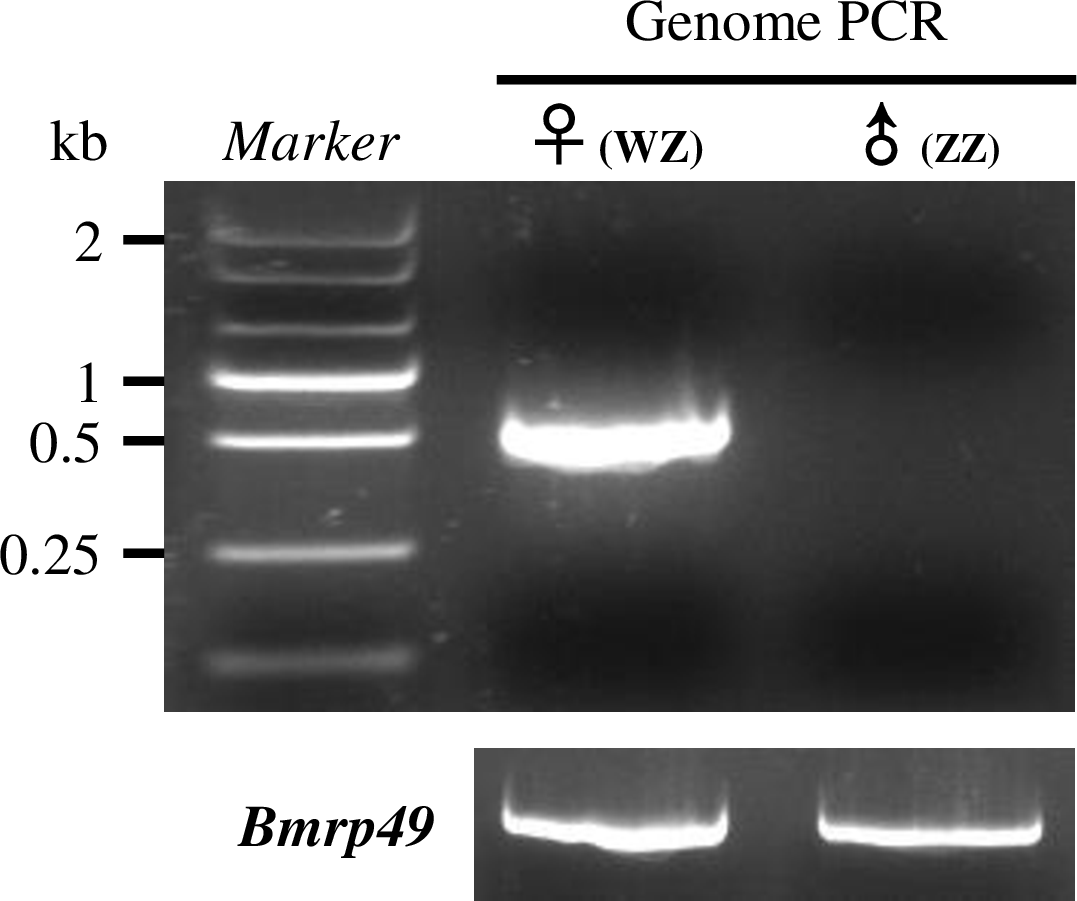

Supplement: S1 Fig — The primer pair flank Fem precursor was used to confirm genotype of mutants and embryos. (TIF) [file pgen.1009194.s003.tif]

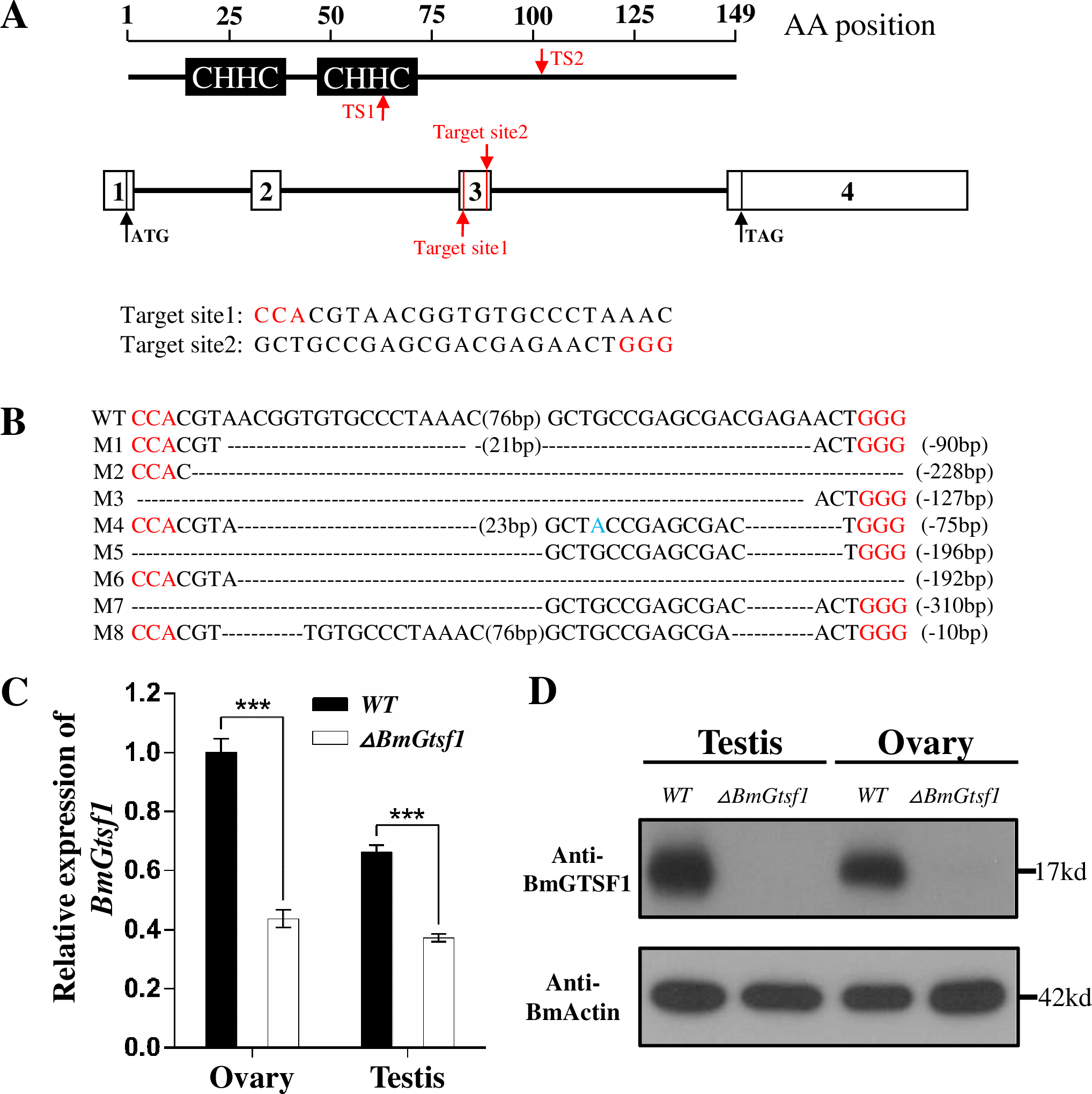

Supplement: S2 Fig — (A) Schematic diagram of the BmGtsf1 gene structure and sgRNA-target sites. The two sgRNA target sites located on the sense strand in exon 3. (B) Diverse types of mutations at BmGtsf1 locus detected by sequencing. The A labelled in light blue means nucleotide replacement. (C and D) The depletion efficiency confirmed by qRT-PCR (C) and western blot (D). (TIF) [file pgen.1009194.s004.tif]

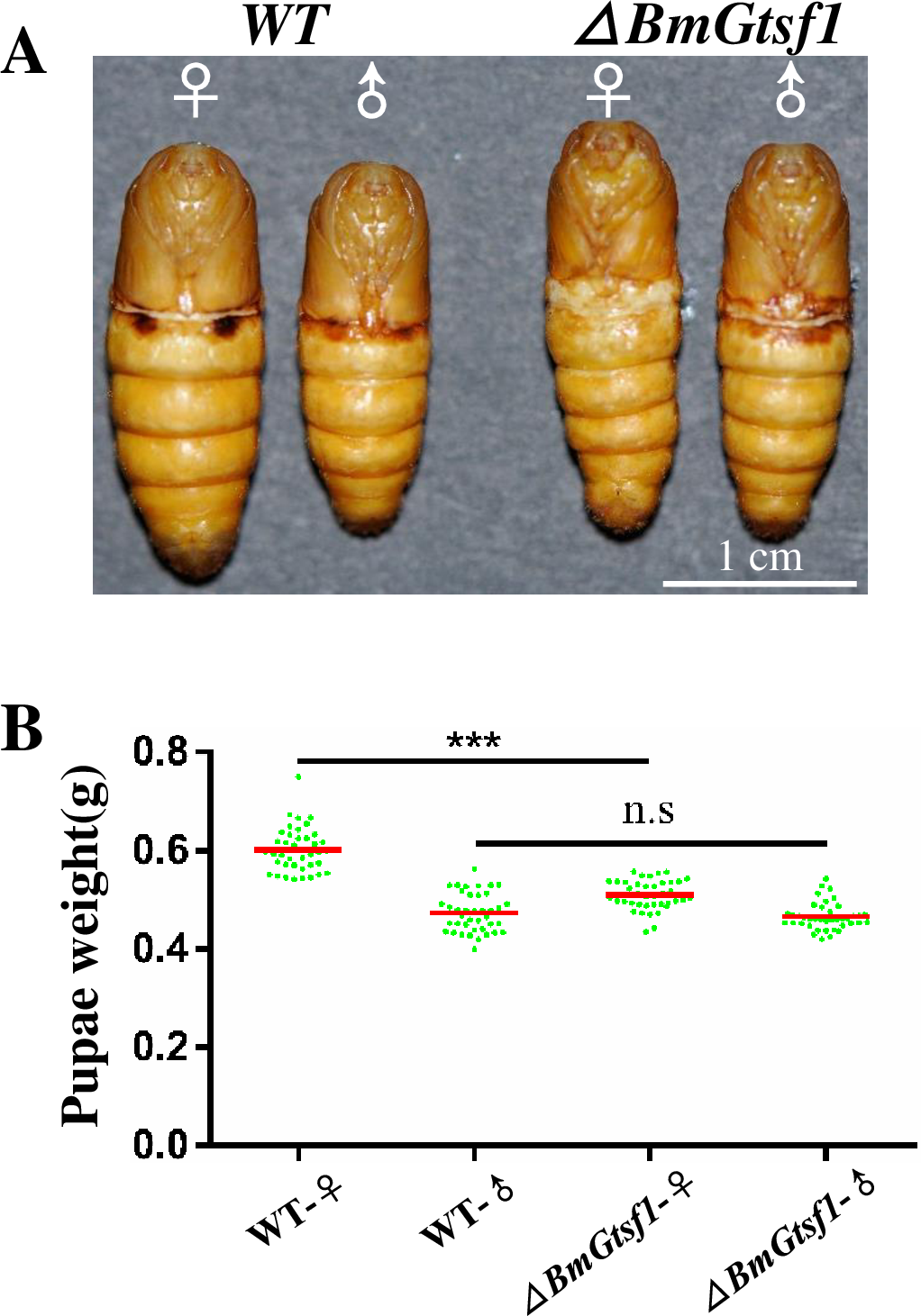

Supplement: S3 Fig — (A) BmGtsf1 female mutant showed smaller body size compared with that of WT. (B) Pupa weight of WT and BmGtsf1 mutants. The red bar is average value, n = 30. The asterisks (***) means p value < 0.001, and n.s means there is no significant difference between the groups. (TIF) [file pgen.1009194.s005.tif]

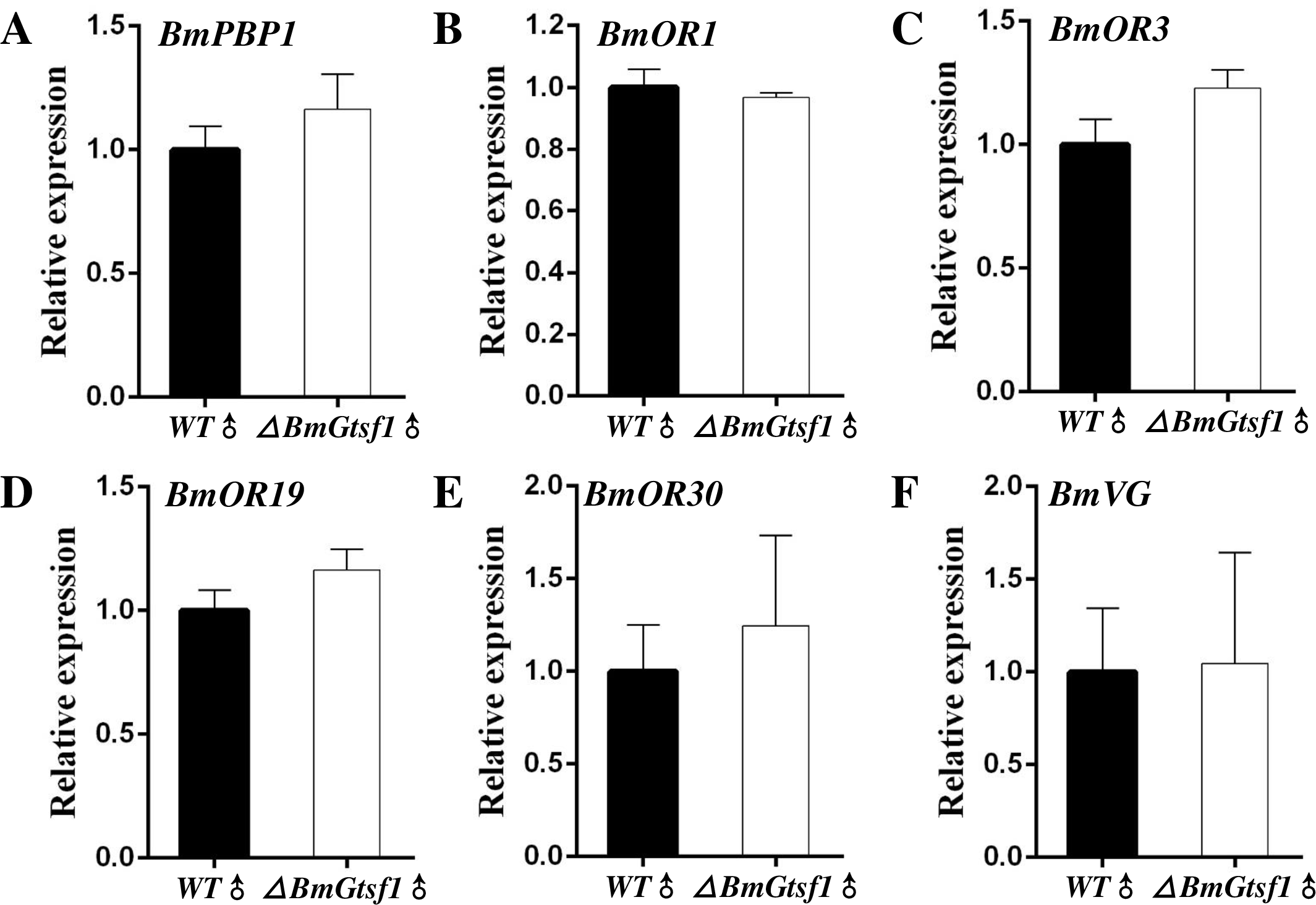

Supplement: S4 Fig — (A-F) Relative expression of BmPBP1 (A), BmOR1(B), BmOR3 (C), BmOR19 (D), BmOR30 (E) and BmVg (F) in BmGtsf1 male mutants. RNA extracted from antenna of adults was used for qRT-PCR analyses in Fig 3C–Fig 3G, while the expression of BmVG was detected in fat body at wandering stage. Error bars are ± SD. (TIF) [file pgen.1009194.s006.tif]

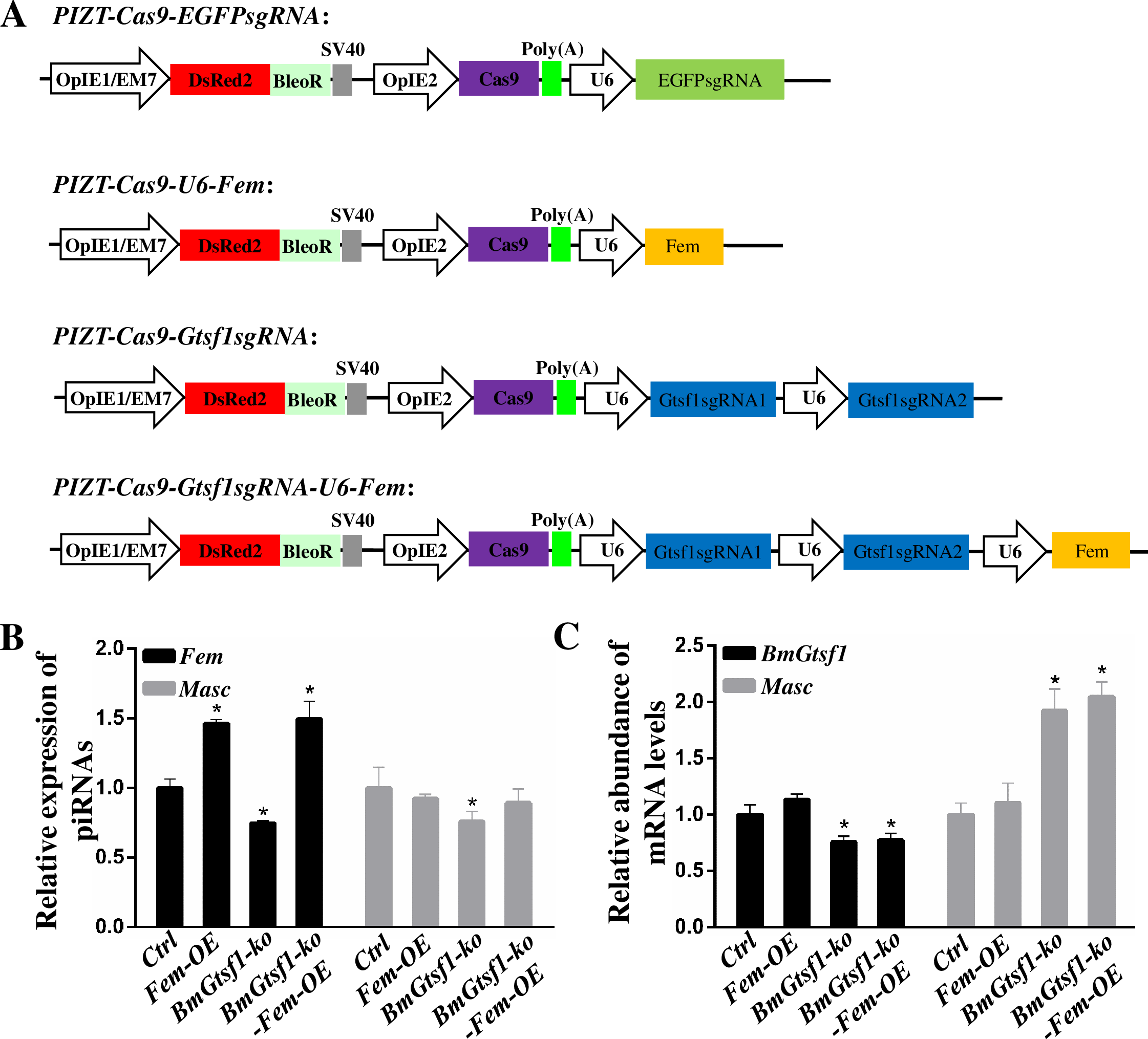

Supplement: S5 Fig — (A) Schematics of the plasmids used in the rescue assay. (B and C) Relative abundance of piRNA abundance (B) and mRNA levels (C) in BmN cells transfected with different plasmids. Error bars are ±SD. The asterisks represent significant differences with p < 0.05. (TIF) [file pgen.1009194.s007.tif]

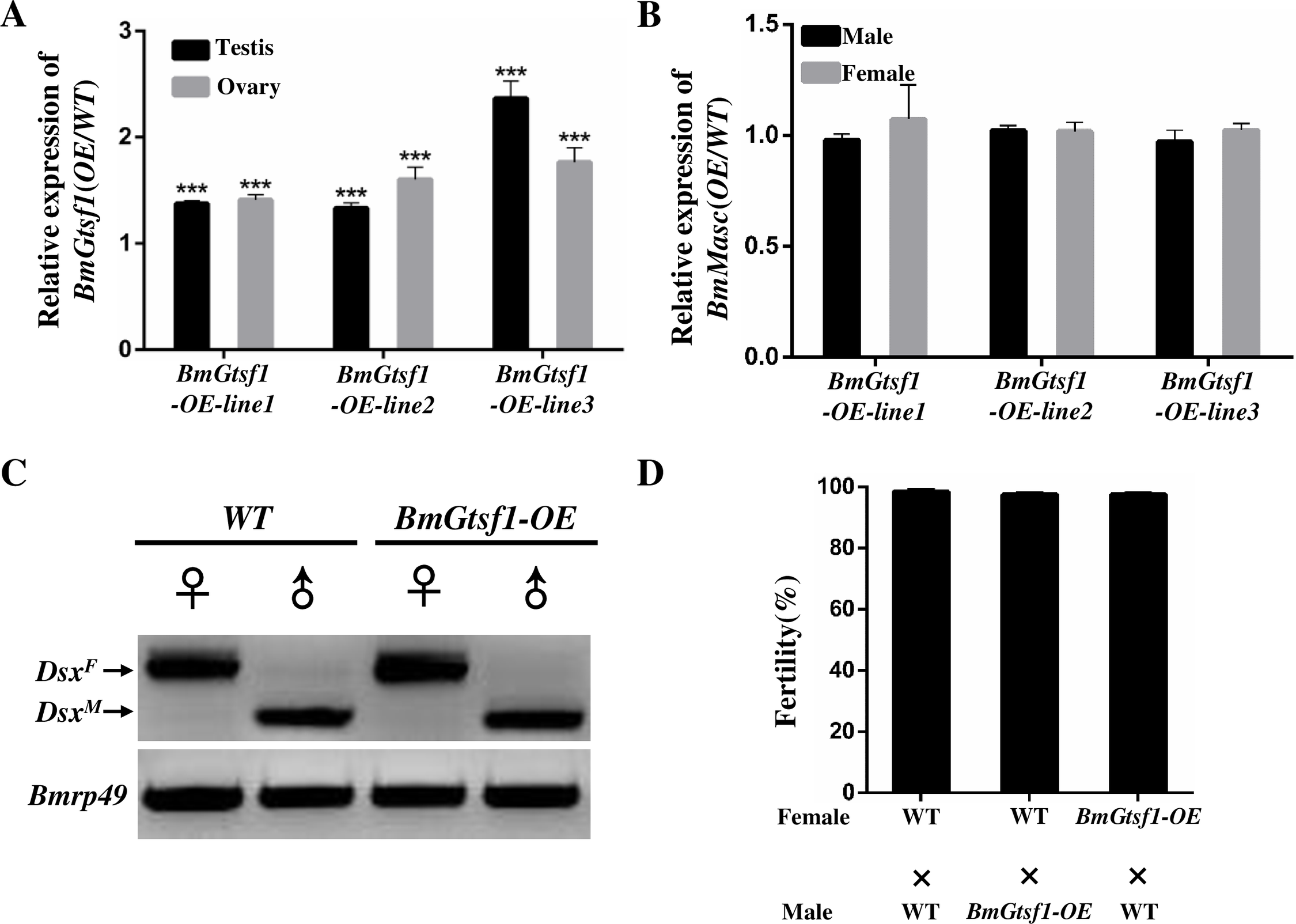

Supplement: S6 Fig — (A and B) Relative expression of BmGtsf1 (A) and BmMasc (B) in overexpression lines. (C) Splicing patterns of Bmdsx in WT and BmGtsf1 overexpression line detected by RT-PCR. (D) Fertility of BmGtsf1 overexpression line, n = 20. RNA extracted from gonads was used for RT-PCR and qRT-PCR analyses. (TIF) [file pgen.1009194.s008.tif]

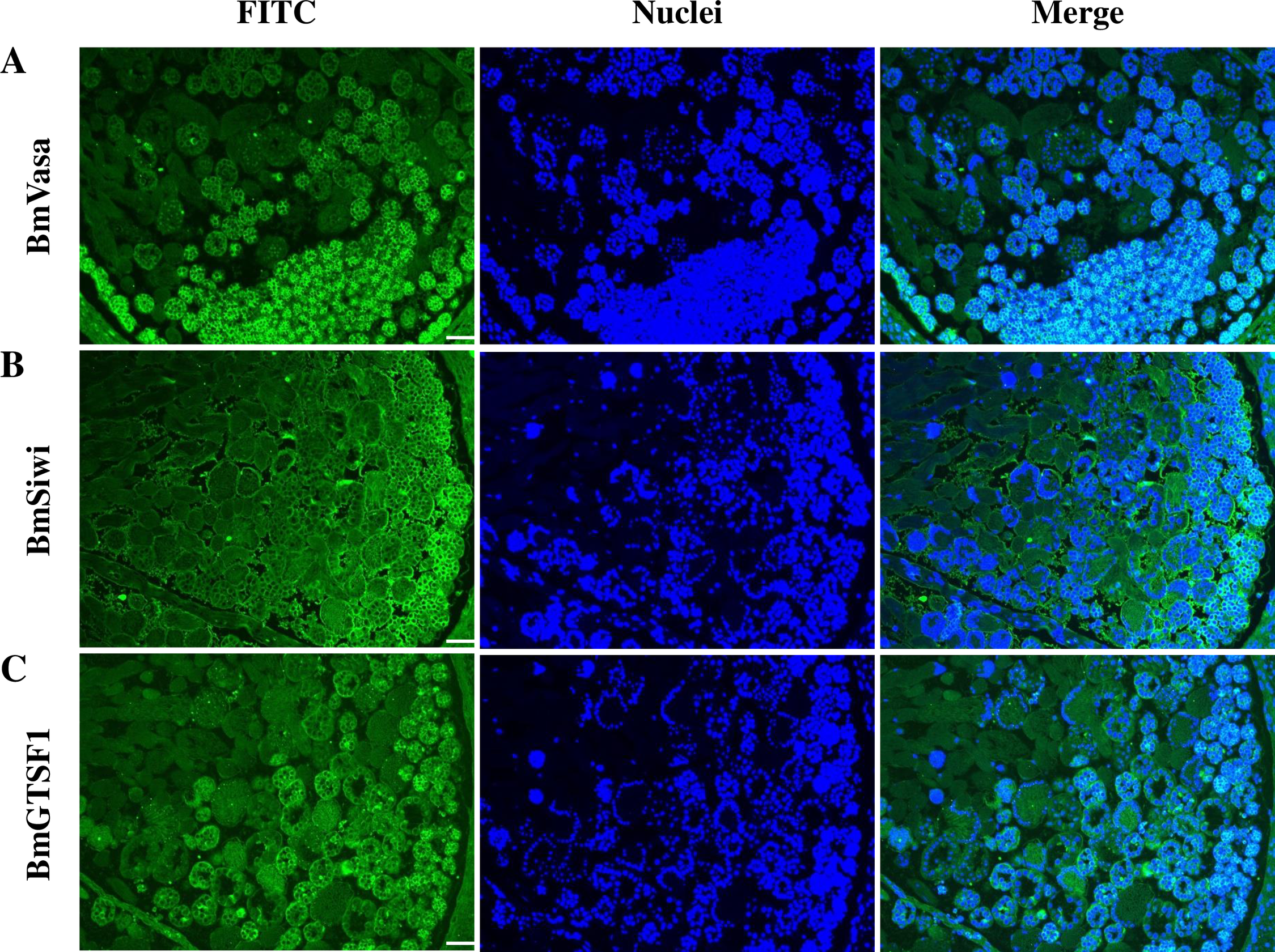

Supplement: S7 Fig — (A-C) Localization of BmVasa (A), BmSIWI (B) and BmGTSF1 (C) in the testes of B. mori. A FITC-conjugated secondary antibody was used for fluorescence detection and Hoechst staining (blue) showed the locations of the nuclei. Scale bar, 50 μm. (TIF) [file pgen.1009194.s009.tif]

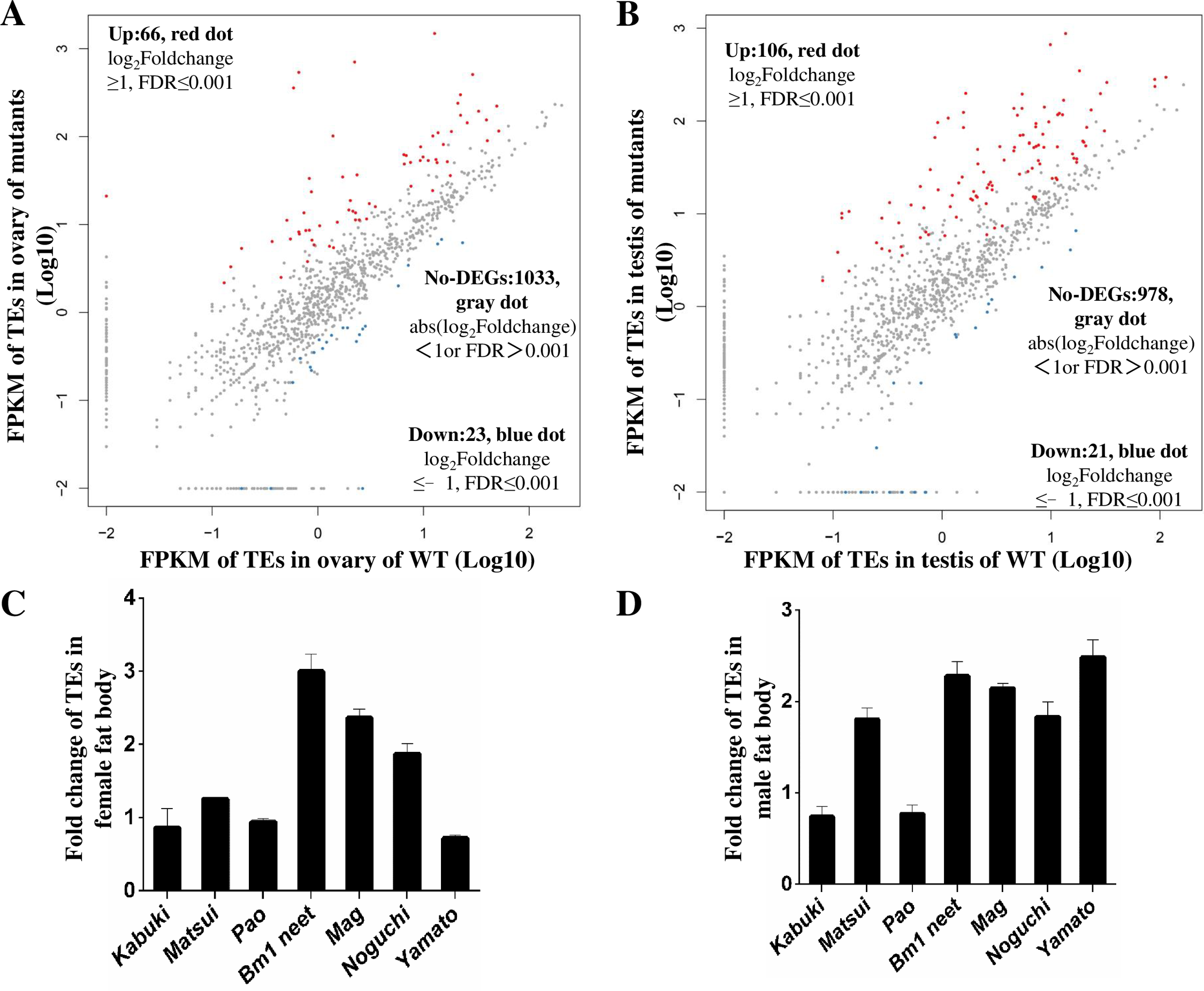

Supplement: S8 Fig — (A and B) RNA-seq analysis of relative transposon levels in ovary (A) and testis (B) of WT and BmGtsf1 mutant. (C and D) Relative mRNA levels of transposon in fat body of female and male mutants. (TIF) [file pgen.1009194.s010.tif]
